# Crystal structure of the γ-hydroxymuconic semialdehyde dehydrogenase from *Pseudomonas* sp. strainWBC-3, a key enzyme involved in para-Nitrophenol degradation

**DOI:** 10.1186/1472-6807-13-30

**Published:** 2013-11-19

**Authors:** Jing Su, Cong Zhang, Jun-Jie Zhang, Tiandi Wei, Deyu Zhu, Ning-Yi Zhou, Li chuan Gu

**Affiliations:** 1 State Key Laboratory of Microbial Technology, School of Life Sciences, Shandong University, Jinan 250100, China; 2 Key Laboratory of Agricultural and Environmental Microbiology, Wuhan Institute of Virology, Chinese Academy of Sciences, Wuhan 430071, China; 3 School of Food & Bioengineering, Qilu University Of Technology, Jinan 250300, China; 4 Sangon Biotech (Shanghai) Co., Ltd, Shanghai 201611, China

**Keywords:** *Pseudomonas* sp*. strain* WBC-3, *para*-Nitrophenol, PNP degradation, γ-hydroxymuconic semialdehyde dehydrogenase, Catalyzing mechanism

## Abstract

**Background:**

*para*-Nitrophenol (PNP) is a highly toxic compound with threats to mammalian health. The *pnpE*-encoded γ-hydroxymuconic semialdehyde dehydrogenase catalyzes the reduction of γ-hydroxymuconic semialdehyde to maleylacetate in *Pseudomonas* sp*. strain* WBC-3, playing a key role in the catabolism of PNP to Krebs cycle intermediates. However, the catalyzing mechanism by PnpE has not been well understood.

**Results:**

Here we report the crystal structures of the apo and NAD bound PnpE. In the PnpE-NAD complex structure, NAD is situated in a cleft of PnpE. The cofactor binding site is composed of two pockets. The adenosine and the first ribose group of NAD bind in one pocket and the nicotinamide ring in the other.

**Conclusions:**

Six amino acids have interactions with the cofactor. They are C281, E247, Q210, W148, I146 and K172. Highly conserved residues C281 and E247 were identified to be critical for its catalytic activity. In addition, flexible docking studies of the enzyme-substrate system were performed to predict the interactions between PnpE and its substrate γ-hydroxymuconic semialdehyde. Amino acids that interact extensively with the substrate and stabilize the substrate in an orientation suitable for enzyme catalysis were identified. The importance of these residues for catalytic activity was confirmed by the relevant site-directed mutagenesis and their biochemical characterization.

## Background

para-Nitrophenol (PNP), which is widely used in the manufacture of medicines, pesticides, dyes, explosives, leather coloring, wood preservatives and rubber chemicals, is highly toxic to animal’s health. Through the respiratory system and skin it affects the blood, liver and central nervous system in the body. It can cause dizziness, rash, itching spam, anemia and various neurological symptoms [1]. It contains a nitroso group in the structure that strongly attracts the electron of phenyl, thus PNP is difficult for degradation in the nature [2].Therefore, a number of studies have been triggered focusing on the microbial degradation of PNP [3-6] and two pathways have been clearly illustrated so far. One is the hydroquinone pathway that is usually found in Gram-negative bacteria. For example, in *Moraxella* sp, PNP monooxygenase converts PNP to hydroquinone via the potential intermediate *p*-benzoquinone [7,8]. The other is the hydroxyquinol (1, 2, 4-trihydroxybenzene) pathway that is preferentially found in Gram-positive bacteria. For example, in *Rhodococcus opacus SAO101*, PNP is converted to hydroxyquinol via 4-nitrocatechol [9,10].

*Pseudomonas* sp. strain WBC-3 utilizes either methyl parathion (O,O-dimethyl O-p-nitrophenol phosphorothioate) or PNP as the sole source of carbon, nitrogen and energy for survival [11]. This bacterium degrades PNP through the hydroquinone pathway. First, PNP is converted to *p*-benzoquinone by PNP 4-monooxygenase (PnpA) and *p*-benzoquinoneis further reduced to hydroquinone by *p*-benzoquinone reductase (PnpB). Next, hydroquinone dioxygenase (PnpCD) converts hydroquinone to γ-hydroxymuconic semialdehyde, which is then converted to maleyacetate by the NAD dependent γ-hydroxymuconic semialdehyde dehydrogenase (PnpE). Finally, maleylacetate reductase (PnpF) catalyzes the conversion of maleyacetate to β-ketoadipate before entering the TCA cycle.

In contrast to the reasonably thorough biochemical and genetic characterization of the PNP degradation, the structural mechanism of each reaction step in the pathway remains unclear. In order to understand the structural basis required for the activity of the enzyme, we embarked on structure determination of these enzymes that catalyze the PNP degradation process.

Recently, the primary X-ray analysis of PnpA in *P.putida DLL-E4* was reported. In which PnpA was crystallized and the diffraction data was obtained [12]. However, there was no further structural report about the enzyme or other molecules involved in the hydroquinone pathway. PnpE belongs to NAD dependent aldehyde dehydrogenase (ALDH) super-family which can catalyze the aldehydes oxidation to corresponding carboxylic acids [13-15]. To date, some crystal structures of NAD (P) ^+^ - dependent ALDH family have been reported [16-20]. These enzymes exhibit wide differences in substrate and cofactor specificity. Sequence alignments of them with PnpE gave amino acid sequence identities ranging from 23% to 45%. Among them mitochondrial aldehyde dehydrogenase from bovine gives the highest sequence identity with PnpE.

This super family involves highly conserved residues Cys which was essential nucleophile and Glu that was the general base necessary to activate Cys for the dehydrogenase reaction. The substrate of PnpE is γ-hydroxymuconic semialdehyde which is unstable in air. So far the catalytic mechanism of this substrate has not been reported. In the current study, we determined the crystal structure of *Pseudomonas* sp. strain WBC-3 apo-PnpE at 2.7 Å resolution and its complex with NAD at 3.1 Å resolution. Through enzyme-substrate docking-guided point mutations, we identified the active site of PnpE and studied its catalytic mechanism.

## Methods

### Bacterial strains, chemicals, media and culture conditions

*Pseudomonas sp. Strain WBC-3 genomic DNA* obtained from Wu Han Institute of Virology, Chinese Academy of Science*. Escherichia coli* strain BL21(DE3) used as expression host, which was cultured at 37°C in lysogeny broth (LB) and transformed as described by Sambrook*et al*. [21]. All chemical products used in the experiment were purchased from Sigma Chemical Co. (St Louis, MO, USA).

### Gene cloning and oligonucleotide-directed mutagenesis sequencing

*pnpE* gene from *Pseudomonas sp. strain WBC-3* was cloned into NdeI and XhoI sites of pET29b (Novagen). Nine PnpE mutants (C281A, E247K, F150A, W157A, H275E, F447A, N149A, F154A, I282D) were produced using the two-step PCR strategy [22] and were confirmed by DNA sequencing.

### Protein expression and purification

The *E.coli* cells were cultured in the LB medium containing 100 μg/mL ampicillin until OD_600_ reached 0.8 and were then induced with 1 mM IPTG overnight at 15°C. The cells were harvested by centrifugation. Cells lysis was achieved by sonication method. After centrifugation at 28,000 × g for 45 min, the supernatant was applied to Ni-NTA column. The His-tagged PnpE was eluted with elution buffer (10 mM Tris–HCl pH 8.0, 100 mM NaCl, and 250mM imidazole). The purification process was then followed by anion exchange on a Source-Q column and finally applied to size-exclusion chromatography with Superdex-200 column.

### Enzyme activity assays

The catalytic activity of PnpE was measured by cascade reactions. The biosynthesis of γ-hydroxymuconic was performed in a total volume of 100 μL system containing 0.1 mM hydroquinone, 5.6μg hydroquinone dioxygenase PnpCD (PnpCD was expressed and purified in the same way as described in section 2.3) and 0.1 mM FeSO4 and 100mM Tris–HCl (pH 8.0) at 25°C. The absorbance of the reaction mixture was monitored at 290 nm until no further decrease was observed (about 10 min). At this point, the product solution can be used as the substrate for the next step to test PnpE activity. The assay of PnpE was performed immediately after the production of γ-hydroxymuconic in 100μL reaction system containing 5.2 μg purified PnpE, 10 μL γ-hydroxymuconic (produced by biosynthesis method), 0.1 mM NAD and 100 mM Tris–HCl (pH 8.0). The reaction was incubated at 25°C for 15 min. PnpE activity was determined by measuring the absorbance increase at 340 nm due to NADH formation.

### Crystallization and data collection

Protein concentration was adjusted to 10 mg/mL before setting up crystallization screens at 20°C. Initially the native crystals were grown from 20% (w/v) PEG3350, 0.2 M KNO_3_ at 20°C using the sitting-drop, vapor-diffusion technique. The crystallization condition for the NAD bound PnpE was 0.1 M Bicine pH 8.5, 20% PEG10000 and 1 mM NAD. Both data of the two kinds of crystals were collected on BL17U beam line at the Shanghai Synchrotron Radiation Facility (Shanghai, China) using a MAR 225 CCD detector. Crystals were equilibrated in a cryoprotection buffer containing 15% glycerol (v/v) plus reservoir buffer and then flash frozen in a 100K nitrogen stream. The diffraction images were processed with HKL2000 [23].

### Structure determination and refinement

The crystal structure was solved by molecular replacement methods using the program Phaser of the CCP4 program suite [24] with bovine mitochondrial aldehyde dehydrogenase (PDB code: 1A4Z) [20] as the search model. The initial phase obtained from molecular replacement was further refined using PHENIX [25]. The model was rebuilt using COOT [26]. Data collection and structure refinement statistics are summarized in Table 1. All the molecular graphics figures were generated with PyMol (http://www.pymol.org). The apo and complex structures have been deposited in the Protein Data Bank with access codes of 4GO3 and 4GO4.

**Table 1:** X-ray data collection and refinement statistics

**Parameters**	**Native**	**NAD**
Data collection		
Space group	P21	P21
a(Å)	83.531	86.911
b(Å )	144.252	154.539
c(Å)	138.288	143.435
α(degree)	90	90
β(degree)	93.590	95.241
γ(degree)	90	90
Resolution (Å)	50-2.7	50-3.1
Unique reflections (outer shell)	86090 (8181)	63089 (5766)
*I/σ* (outer shell)	14.5 (3.6)	17.0 (2.8)
*R*_sym_ (outer shell)	0.086 (0.312)	0.054 (0.390)
Refinement		
Resolution range (Å)	50-2.7	50-3.1
Number of reflections (|F| > 0)	83501	60632
*R*_work_	0.2249	0.2067
*R*_free_	0.2366	0.2507
Total number of atoms	29694	29983
RMSD		
Bond length (Å)	0.02	0.011
Bond angles (degree)	1.559	1.555
Ramachandran (%)		
Most favored	92.57%	91.05%
Generously allowed	6.79%	8.35%
Disallowed	0.64%	0.60%

a *R*_sym_ = ∑ _*hkl*_  ∑ _*i*_|*I*(*hkl*)_*i*_ ‒ < *I*(*hkl*) > |/∑_*hkl*_ ∑ *I* < *I*(*hkl*)_*i*_ > over *i* observations.

### Flexible docking of substrate to PnpE

AUTODOCK 4.2 [27] was used to carry out the PnpE-substrate flexible docking. Three out of the nine bonds of the substrate molecule γ-hydroxymuconic semialdehyde were set rotatable. A grid box with sufficient margins (40 × 36 × 36 Å), which enveloped the potential active region of PnpE, was placed to restrain the substrate molecule. This potential active region was implicated by the structure of *E.coli* L-Lactaldehyde dehydrogenases (PDB code: 2imp) [28], which is homologous with PnpE. In the active region of PnpE, 9 residues (C281, E247, N149, F150, F154, W157, H275, F447, I282) were assigned as flexible. A genetic search algorithm [27] was used for the docking and a total of 2,500,000 steps of energy evaluations were performed during the docking. Finally, the first ranked docking result according to the interaction energy was chosen as the result. The flexible docking was performed on a 12-core computer station, which consists of Intel Itanium2 Dual Core running at 1.6 GHz. The average wall clock for one docking run was about 2 hours.

## Results and discussion

### Overall structure of apo-PnpE

**Figure 1:**
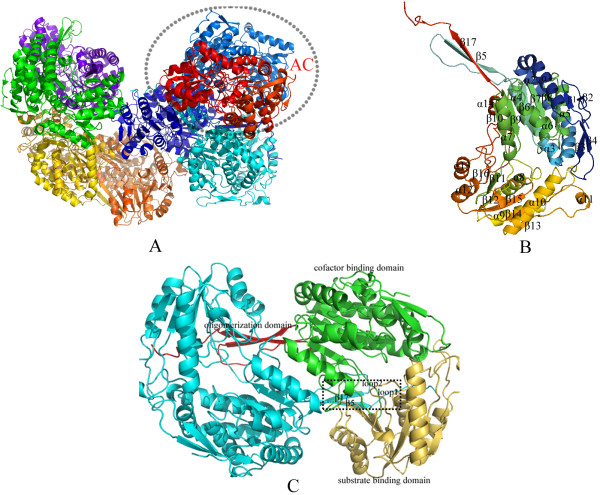
**Overall structure of PnpE. A.** The eight PnpE monomers are represented with cartoon in different colors. AC, BF, DG and EH form four homodimmers. The dashed region denotes one dimer AC. **B.** The secondary structures of PnpE monomer are depicted in rainbow colored cartoon. 17 β –strands and 14 α-helices are labeled. **C.** Structure of PnpE dimer. The dimer is shown in cartoon model. Molecular A is in blue color and molecular C is shown in three colors with each represents one domain: the oligomerization domain in red color, the cofactor binding domain in green color and the substrate binding domain in yellow color. The dash region denotes the interface areas that mediate dimerization.

The crystal belongs to space group P21. The asymmetric unit contains eight monomers named A, B, C, D, E, F, G and H which form four homodimers (Figure 1A). The monomer of PnpE has the typical structural organization βαβ of the ALDH family, with 17 β-strands (β1-β17) and 14 α-helices (α1-α14) (Figure 1B). These secondary structures belong to three domains (Figure 1C): a substrate binding domain, a cofactor binding domain, and an oligomerization domain mediating protein dimerization.

The substrate binding domain consists of a central six-stranded β-sheet (β11 to β16) and six α-helices (α8 to α13), while the cofactor binding domain contains a nine-stranded β-sheet (β1-β4) (β6-β10) and seven α-helices (α1-α7) (Figure 1B).

The four homodimers show high structural similarities. Structure comparisons between AC and other three homodimmers give root mean square deviation (rmsd) values of 0.267 Å, 0.326 Å and 0.214 Å for C_α_ respectively. The dimer interface consists of β5 (residues F130 to K137), β17 (E469 to N476), loop1 (D117 to F130) and loop2 (V477 to R487) (Figure 1B). Loop1 connects β5 and α3. Loop2 is sited in the C terminal. The interface area of the PnpE monomer for dimerization is 3228.7 Å.

Among all the structure-known homologues of PnpE, the bovine aldehyde dehydrogenase [20] shares the highest sequence identity (45%) with PnpE. The human aldehyde dehydrogenase [16] follows close behind with a sequence identity of 44%. The result of sequence alignment indicates these proteins may share similar structures. Structure superimposition shows bovine aldehyde dehydrogenase has a rmsd of 0.925 Å for C_α_ with PnpE, meanwhile the C_α_ rmsd between human aldehyde dehydrogenase and PnpE is 0.943 Å.

### Structure of PnpE-NAD complex

**Figure 2:**
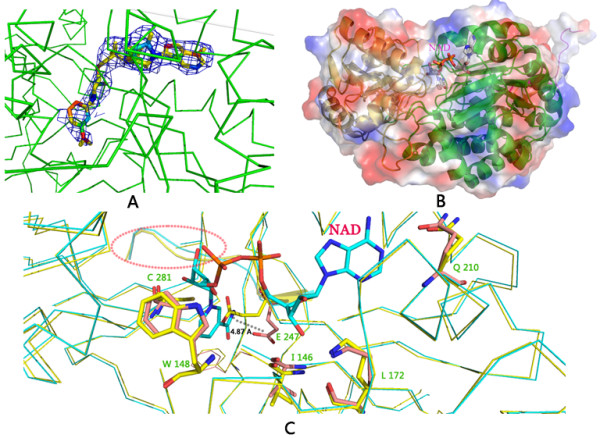
**Structure of PnpE-NAD complex. A.** The difference electron density map (2F*o*-F*c*) calculated at 3.1 Å resolution using phase from the final model with NAD and contoured at 1.0σ reveals the existence of NAD with faily electron density within molecule **A. B.** PnpE cofactor and substrate binding domains. The yellow cartoon stands for the substrate binding domain and the green cartoon represents the cofactor binding domain. NAD is shown in sticks. **C.** Structure comparison of the apo and NAD bound PnpE. Yellow ribbon represents the apo-PnpE structure; Green ribbon stands for the NAD bound structure. The NAD is shown in color sticks. Six amino acids (C281, E247, Q210, W148, I146 and K172) interact with the cofactor. These amino acids are shown in stick. The dash region has the most significant structure discrimination and E247 moves the most.

There are also eight PnpE-NAD complexes in asymmetric unit. NAD molecule was found in all PnpE monomers (Figure 2A). The cofactor binding domain of PnpE is gathered together like a clamp that can hold the NAD (Figure 2B).

In the PnpE-NAD complex structure, NAD is situated in a cleft of PnpE. The cofactor binding site is composed of two pockets. The adenosine and the first ribose group of NAD bind in one pocket and the nicotinamide ring in the other. There are several hydrogen bond interactions between PnpE and NAD. The adenine N1A amino group of NAD forms a hydrogen bond with OE1 of Q210 of PnpE. The ribose hydroxyl groups O2B and O3B receive hydrogen bonds from the side chain of K172 and V146, respectively. The diphosphate O2N donates a hydrogen bond to W148. The C4N accepts a hydrogen bond from C281. N7N accepts a hydrogen bond from E247 (Figure 2C).

The PnpE structure of the PnpE-NAD complex is quite similar to apo-PnpE with a C_α_ rmsd of 0.52 Å. The most significant structural discrimination is observed at the loop between L246 and G252, in which the spatial position of the E247 moved 4.87 Å. This segment belongs to NAD binding region. In the apo-PnpE structure, this loop blocks the NAD binding site. In the complex structure, however, it moves to open the binding site to create enough space for NAD binding (Figure 2C).

### Structural bases for the specificity of cofactor binding

**Figure 3:**
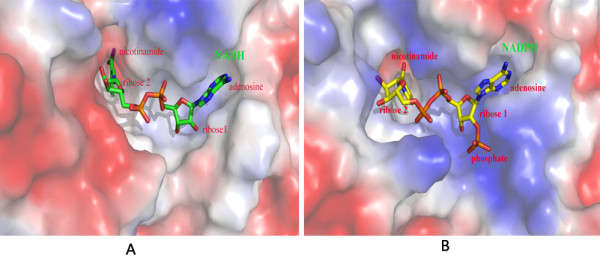
**Comparison of the cofactor binding pockets of PnpE and BADH (PDB code: 2wme) **[29]**in electrostatic surface representations.** Red represents negative charge and blue represents positive charge. **A.** Cofactor binding pocket of PnpE. NAD is shown in sticks. **B.** Cofactor binding pocket of BADH. NADP is depicted in sticks.

So far, both NAD and NADP have been known to be cofactors of the ALDH family. PnpE specifically uses NAD as cofactor. In order to elucidate the structural basis for the coenzyme specificity of PnpE, we compared structures of PnpE-NAD and NADP bound Betaine Aldehyde Dehydrogenase (BADH) (PDB code: 2wme) from *Pseudomonas Aeruginosa*[29] (Figure 3). Among NADP dependent proteins with known structures, BADH shares the highest sequence identity of 41% with PnpE. Since the only difference between NAD and NADP is that the later has an extra phosphate group, PnpE and BADH should be able to distinguish them by this point. Both PnpE and BADH contain a two-pocket NAD (P) binding site. The first pocket is close to the molecule surface and accommodates the adenosine moiety of NAD, whereas the second, which is deeply located in the active site, can accommodate the nicotinamide ribose moiety. From the structures we can see clearly that the second pocket of BADH is wider than that of PnpE. This means BADH has extra space to accommodate the phosphate group of NADP whereas PnpE does not. On the other hand, in BADH the cofactor bind pocket surface around phosphate group is highly positive charged to match the negative charge of the phosphate group. This is not the case in PnpE. Therefore, PnpE can only use NAD rather than NADP as its cofactor.

### Molecular docking results and mutagenesis analysis

**Figure 4:**
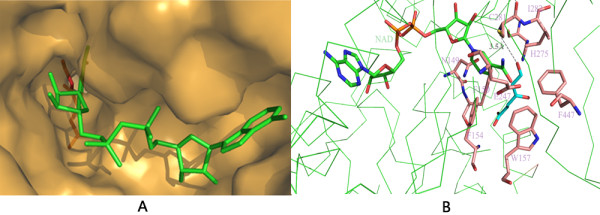
**Substrate binding pocket predicted with the autodock result. A.** PnpE is shown in surface model in yellow. NAD is depicted in green sticks and γ-hydroxymuconic semialdehyde is shown in red sticks. **B.** PnpE is shown in green ribbon model. There are nine amino acids interact with the substrate: C281, E247, F150, I282, F154, H275, F447, W157 and N149.

The substrate γ-hydroxymuconic semialdehyde for PnpE contains an enol structure, which tends to convert into keto under aerobic condition. So it is impracticable to get holo-PnpE structure. To figure out the catalytic mechanism of PnpE without holo-PnpE structure we performed flexible docking for NAD bound PnpE and γ-hydroxymuconic semialdehyde using AUTODOCK (experimental procedure detailed in section 2.7). The docking result is illustrated in Figure 4. The substrate located in a narrow cleft close to NAD. There are nine residues (C281, E247, F150, I282, F154, H275, F447, W157, N149) probably involved in the substrate binding. C281 forms hydrogen bond with the aldehyde group of the substrate directly and may play a key role in the enzymatic reaction. F150, F154, F447, W157, N149, I282 and H275 probably composed a hydrophobic pocket to accommodate the substrate. However, W157 and F447 are a little farther from the substrate compared to other five residues.

**Figure 5:**
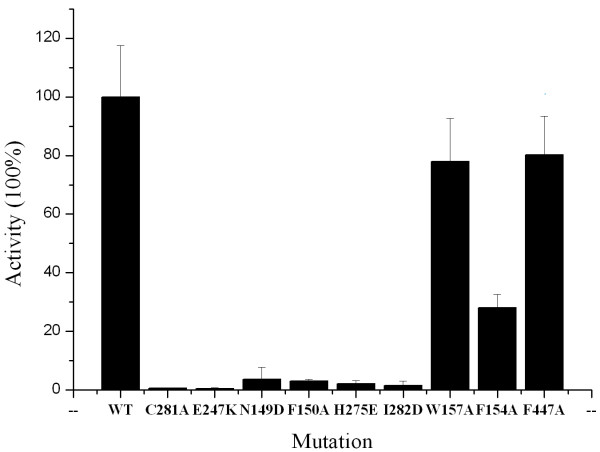
**Enzyme activities of wild type PnpE and different mutants.** The activity of the wild type is set to 100%. C281A, E247K, N149D, F150A, H275E, I282D, W157A, F154A, F447A are mutants made according to docking results.

To confirm the molecular docking result, nine single amino acid mutants (C281A, E247K, N149D, F150A, F154A, W157A, H275E, F447A, I282D) were generated using a two-step PCR strategy. Their enzyme activities were then measured and repeated four times (Figure 5). The results showed the mutants of C281A, E247K, N149D, F150A, H275E and I282D completely lost their activity. The activity of F154A decreased about 60%. The activity of W157Aand F447A decreased about 20%. C281 and E247, which are highly conserved across the ALDH family members, have been known to play essential roles in the catalytic process. The loss of PnpE activity by N149D, F150A, H275E, I282D, and the significant decrease of the activity by F154A also argue that these amino acids play important roles in the substrate binding. W157 and F447 do not contact substrate directly, thus W157A and F447A only affected enzymatic activity modestly, compared to the significant effects of other seven mutations. These data are consistent with the result of AUTODOCK calculations.

## Conclusions

**Figure 6:**
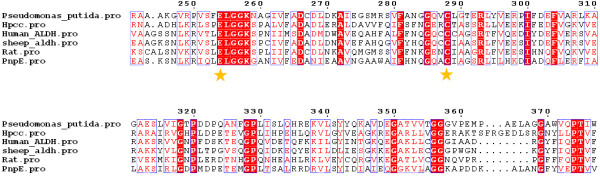
**Multiple sequence alignment of PnpE with Human mitochondrial Aldehyde Dehydrogenase (PDB entry 1cw3) **[33]**, rat 10′formyltetrahydrofolate dehydrogenase (PDB entry 2o2q) **[34]**, sheep Aldehyde Dehydrogenase (PDB entry 1bxs) **[17] ,***Pseudomonas putida *BADH (PDB entry 2wme) **[29]**and *Thermus thermophilus *Hpcc (PDB entry 2d4e).** The highly conserved catalytic residues are labeled with red asterisks.

**Figure 7:**
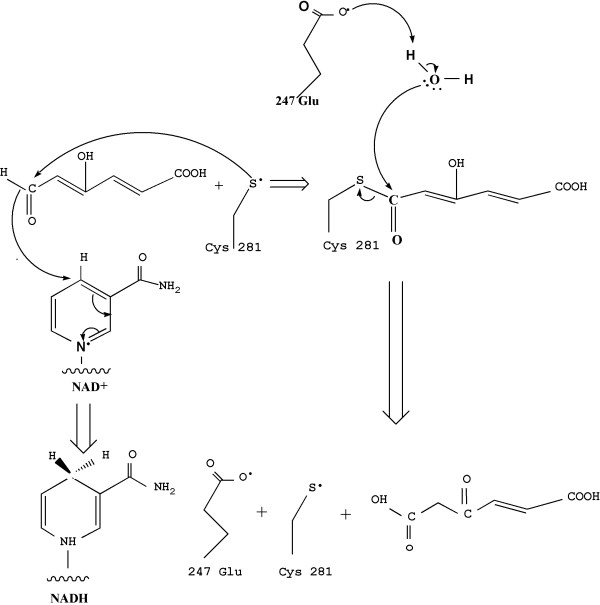
The oxidation process of γ-hydroxymuconic semialdehyde to maleyacetate with NAD as cofactor by PnpE.

Sequence-based alignment of PnpE and previously determined structures of ALDHs shows that two residues (E247 and C281) are highly conserved across different species (Figure 6). These two amino acids act as the catalytic residues that transport electron, according to the mechanism elucidated in other homologous proteins [30-32]. In this regard, PnpE may have a similar catalytic mechanism (Figure 7). The catalytic C281 plays a role as a nucleophilic reagent. It attacks the hydroxymuconic semialdehyde substrate and forms a covalent intermediate. Next, the hydride from this intermediate transfers to NAD to form NADH and the thioacyl enzyme intermediate. The other catalytic residue E247 directly attacks the acyl-sulfur bond and releases the acid product prior to NADH dissociation.

### Accession codes

The atomic coordinates and structure factors have been deposited under the accession codes: 4GO3 and 4GO4 in the Protein Data Bank, Research Collaboratory for Structural Bioinformatics, Rutgers University, New Brunswick, NJ (http://www.rcsb.org/).

## Competing interests

The authors declared that they have no competing interests.

## Authors’ contributions

JS carried out gene clone, protein purification, crystallization, data measurement of PnpE and PnpE-NAD. CZ performed oligonucleotide-directed mutagenesis and purification. JJZ carried out enzyme activity assays. TDW performed docking of substrate to PnpE. DYZ refined the structure of PnpE and PnpE-NAD. LCG conceived of the study and designed the experiments and prepared the manuscript. NYZ prepared the manuscript. All authors read and approved the final manuscript.
